# Chemopreventive Effect of *Cratoxylum formosum* (Jack) ssp. *pruniflorum* on Initial Stage Hepatocarcinogenesis in Rats

**DOI:** 10.3390/molecules26144235

**Published:** 2021-07-12

**Authors:** Piman Pocasap, Natthida Weerapreeyakul, Rawiwan Wongpoomchai

**Affiliations:** 1 Department of Pharmacology, Faculty of Medicine, Khon Kaen University, Khon Kaen 40002, Thailand; pimapo@kku.ac.th; 2 Human High Performance and Health Promotion Research Institute, Khon Kaen University, Khon Kaen 40002, Thailand; 3 Division of Pharmaceutical Chemistry, Faculty of Pharmaceutical Sciences, Khon Kaen University, Khon Kaen 40002, Thailand; 4 Department of Biochemistry, Faculty of Medicine, Chiang Mai University, Chiang Mai 50200, Thailand; 5 Research Center for Development of Local Lanna Rice and Rice Products, Chiang Mai University, Chiang Mai 50200, Thailand

**Keywords:** *Cratoxylum formosum* ssp. *pruniflorum* (Kurz) Gogelein, hepatocarcinogenesis, diethylnitrosamine, rat, immunohistochemistry

## Abstract

*Cratoxylum formosum* ssp. *pruniflorum* (Kurz) Gogelein (CP) is an indigenous plant found mainly in southeast Asia. Several in vitro studies have confirmed its activity against hepatocellular carcinoma; however, in vivo studies of the effect of CP on liver cancer are needed. This study investigated the effect of CP on early-stage hepatocarcinogenesis in rat liver when using diethylnitrosamine (DEN) as a carcinogen. Immunohistochemistry was used to detect (a) upregulation of glutathione *S*-transferase placental (GST-P) positive foci, (b) the proliferating cell nuclear antigen PCNA, and (c) apoptotic cells in the liver as indicators of early-stage carcinogenesis. Immunohistochemical parameters were observed in rats given CP orally following DEN injection. Rats given DEN presented overexpression of GST-P positive foci, PCNA, and apoptotic cells, indicating the formation of cancerous tissues, and these effects were diminished by CP treatment. CP thus inhibited hepatocarcinogenic effects in an animal model. These results could help plan further in vivo studies and support the use of CP to prevent processes that promote the pathogenesis of hepatocellular carcinoma in humans.

## 1. Introduction

*Cratoxylum formosum* ssp. *pruniflorum* (Kurz) Gogelein (CP) (“Tiew kon” in Thai) [1] is a plant in the Guttiferae family widely distributed in Southeast Asia [2]. The indigenous plant is used locally as an ingredient in traditional cuisines and remedies [3] and the main ingredient in bitter nail tea in Yunnan province, China, and Vietnam [4]. Studies indicate that the phytochemical constituents—e.g., xanthones, flavonoids, and triterpenoids—in CP could be used for therapeutic purposes [4,5,6]. Several related studies examined the potency of CP for various possible therapeutic uses such as antibacterials [7], anti-inflammatories [8], gastroprotective effects [9], and prevention of Alzheimer’s disease [10]. Another promising application of CP is its potential for anticancer activity.

Cancer is the leading cause of death worldwide. In 2020, 9.9 million people were estimated to have died from cancer [11]. Hepatocellular carcinoma (HCC) is one of the most pernicious cancers due to its high mortality, ranking as the fourth leading cause of cancer death and increasing [11]. In terms of geography, the prevalence and mortality rate of HCC in southeast Asia is twice the worldwide values, ranking second among cancer-related deaths in the region [12], requiring urgent countermeasures. The use of medicinal plants that are easily found in the region with potential anticancer activity is a reasonable course of action, not as a definitive treatment but as an adjunct. The primary burden is not only the resistant nature of HCC—which is refractory to most currently available anticancer drugs [13]—but also lack of awareness is an important barrier to early diagnosis due to the absence of specific symptoms in the early stages [14], so a preventive strategy should be prioritized.

We previously reported the chemotherapeutic property of CP against HCC in vitro. CP exerts an anticancer activity through several mechanisms, including DNA alkylation, suppressing DNA supercoil relaxation, and overexpression of the death receptor TRAILR5 [15]. These triggers lead to apoptotic cell death in extrinsic and intrinsic pathways, as indicated by various caspase cascade activations [15,16]. Our previous phytochemical analysis indicated that the hydroethanolic extract contained several phytochemical constituents, including xanthones, phenolic compounds (e.g., gallic acid, chlorogenic acid, and caffeic acid), and fatty acids (e.g., palmitic acid and oleic acid) [10,15]. Those results agreed with other studies in which the group of compounds (including xanthones and phenolics) represents the major phytoconstituents of CP, so could contribute to the observed chemotherapeutic properties [17,18,19]. Several studies have used different cell lines exhibiting the anticancer capability of CP [17,20]. To our knowledge, there has been no study using an animal model to determine the potency of CP against cancer. This study was, therefore, conducted to examine the efficacy of CP in carcinogen-induced rats. While our previous study demonstrated CP’s chemotherapeutic properties (as a suppressing agent) to eradicate cancer in vitro, the present study focused on its chemopreventive activity (as a blocking agent) to prevent the initial steps of hepatocarcinogenesis in vivo. The outcome of the study could support the use of CP as an herbal adjuvant of anticancer therapeutics and be a baseline for further study and use in humans.

## 2. Results

### 2.1. Effects of CP on Body/Internal Organ Weights and Food/Water Consumption

**Figure 1:**
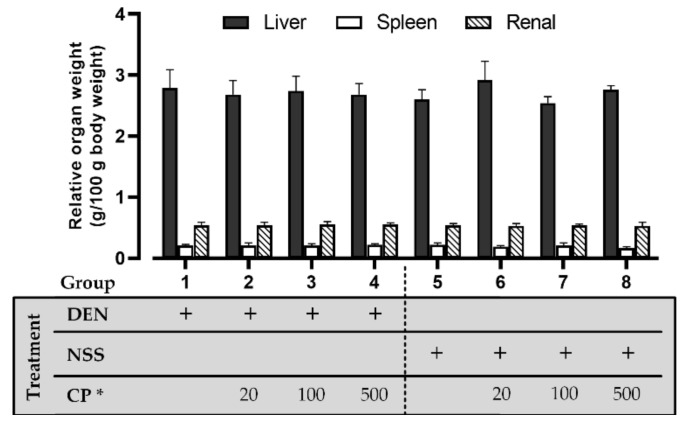
The effect of CP on relative vital organ weight. DEN or NSS were initially administered at week 0 and 1. Then either DW (for groups 1 and 5) or CP were fed weekly starting from week 2 till the end of the experiment. Data was distributed normally (Shapiro-Wilk and Levene’s test: *p* ≥ 0.05) and was analyzed for statistically significance using one-way ANOVA with LSD’s post hoc test: F_(Liver)_ = 1.577 (*p* = 0.159), F_(Spleen)_ = 1.992 (*p* = 0.070), and F_(Renal)_ = 0.549 (*p* = 0.794). * CP dosage unit; mg/kg BW.

Results show no significant effect of CP on body growth as observed among each treatment group. The body weights, as well as food/water consumption in the CP-treated groups, were not significantly different from the initial DEN-induced (Group 1) or the NSS (normal saline)-injected group (Group 5) (Table 1). A significant difference was also not detected in the relative weight of the livers, kidneys, or spleens in each treatment group (Figure 1), implying there was no effect of the CP extract at the treatment regimen on the observed vital organs.

### 2.2. Effects of CP on ALT and AST Levels

**Figure 2:**
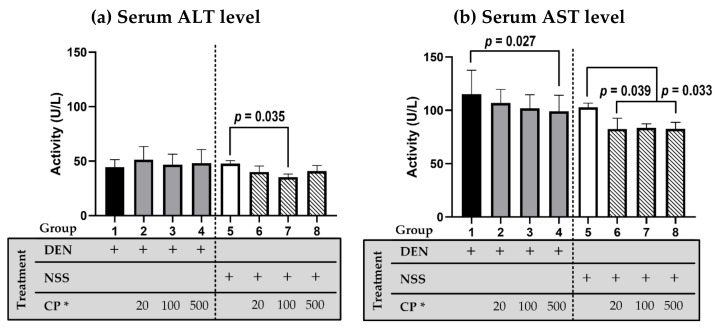
The effect of CP on the serum level of ALT (**a**) and AST (**b**). DEN or NSS were initially administered at week 0 and 1. Then either DW (for groups 1 and 5) or CP were fed weekly starting from week 2 till the end of the experiment. Data was distributed normally (Shapiro–Wilk and Levene’s tests: *p* ≥ 0.05) and was analyzed for statistically significant using one-way ANOVA with LSD’s post hoc test: F_(ALT__)_ = 2.243 (*p* = 0.048) and F_(AST__)_ = 5.061 (*p* < 0.001). * CP dosage unit; mg/kg BW.

The rat serum ALT and AST were monitored (Figure 2). No significant difference in ALT serum level was observed amongst the treatment groups, except CP extract at 100 mg/kg BW, which lowered the ALT level compared to the NSS-injected group (Figure 2a). The AST serum levels were decreased in the CP extract treatment groups (20 and 500 mg/kg) compared to NSS injected group. The CP extract at 500 mg/kg BW reduced the elevated AST serum level induced by DEN (Figure 2b). Overall, the data indicate no hepatoxicity of CP at the regimen used.

### 2.3. Effect of CP on GST-P Positive Foci

**Figure 3:**
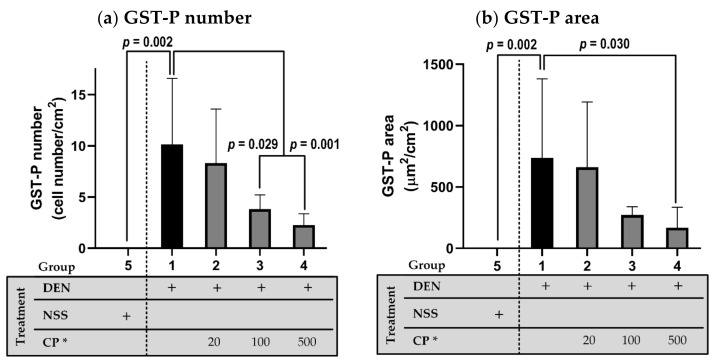
The effect of CP on GST-P positive foci formation as represented by a number (**a**) and area (**b**). DEN or NSS were initially administered at week 0 and 1. Then either DW (for groups 1 and 5) or CP were fed weekly starting from week 2 till the end of the experiment. Data displays non-normal distribution (Shapiro–Wilk and Levene’s test: *p* < 0.05) and was analyzed for statistical difference using nonparametric Mann–Whitney U test. * CP dosage unit; mg/kg BW.

The initial administration of DEN induced hepatocarcinogenesis, as indicated by the GST-P positive foci, whereas no positive trait was observed in the NSS-injected group. GST-P positive foci expression—as indicated by GST-P number (Figure 3a) and GST-P area (Figure 3b), initiated by DEN injection—was significantly reduced after CP extract treatment (100 and/or 500 mg/kg BW). It is evident that CP potentially prevents or attenuates hepatocarcinogenesis.

### 2.4. Effect of CP on PCNA Expression

**Figure 4:**
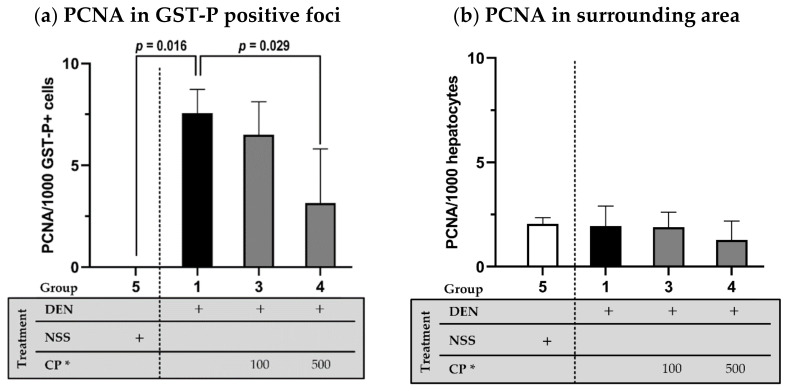
The effect of CP on PCNA expression in GST-P positive foci area (**a**) and surrounding area (**b**). DEN or NSS were initially administered at week 0 and 1. Then either DW (for groups 1 and 5) or CP were fed weekly starting from week 2 till the end of the experiment. Data displays non-normal distribution (Shapiro-Wilk and Levene’s test: *p* < 0.05) and was analyzed for statistical difference using non-parametric Mann–Whitney U test. * CP dosage unit; mg/kg BW.

The ability of CP to retard hepatocarcinogenesis was confirmed by PCNA expression monitoring. The effective doses that statistically decreased GST-P positive foci (100 and 500 mg/kg BW) were further determined. The number of proliferating cells—as indicated by PCNA expression in the GST-P positive foci area (the vicinity of carcinogenesis)—was halted because of the CP extract treatment (500 mg/kg BW) (Figure 4a). The CP extract also displayed no mutagenicity since there was no difference in PCNA expression in the surrounding area of any of the CP treatments (Figure 4b).

### 2.5. Effect of CP on Apoptosis Induction

**Figure 5:**
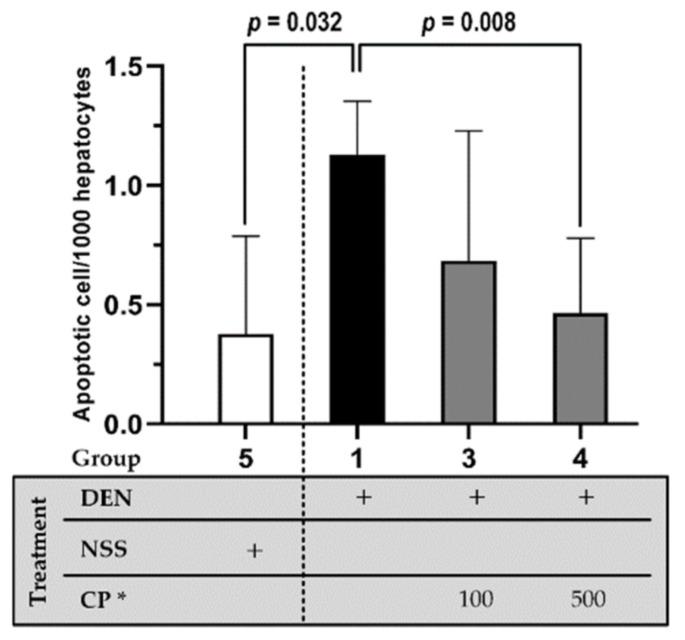
The effect of CP on apoptotic cell expression as indicated using TUNEL assay. DEN or NSS were initially administered at week 0 and 1. Then either DW (for groups 1 and 5) or CP were fed weekly starting from week 2 till the end of the experiment. Data displays non-normal distribution (Shapiro-Wilk and Levene’s test: *p* < 0.05) and was analyzed for statistical difference using nonparametric Mann Whitney U test. * CP dosage unit; mg/kg BW.

The apoptotic cell indicated by TUNEL positive was consequently determined as a marker of carcinogenesis progress. DEN-induced rats exhibited an increased number of apoptotic cells in liver tissue than those initially injected with NSS. Enhanced apoptotic expression—due to DEN induction—was reduced by CP extract (500 mg/kg BW) (Figure 5), indicating CP’s efficacy to suppress the progress of carcinogenesis in rat liver. The CP extract displayed promising efficacy to prevent early-stage hepatocarcinogenesis as it reduced several parameters that are hallmarks of carcinogenesis progression.

## 3. Discussion

In the current study, hepatocarcinogenesis was initiated using the carcinogen diethylnitrosamine (DEN). After intraperitoneal administration, DEN is metabolized by cytochrome P450 (CYP2E1) in the liver, yielding an active mutagenic metabolite that can directly bind to nucleic acids, resulting in DNA mutation and cancer formation [21]. The reactive metabolite also produces reactive oxygen species that induce liver inflammation, leading to hepatocellular carcinoma [22]. The early stage of hepatocarcinogenesis is detected later by observing GST-P positive foci formation in the vicinity [23], which is overexpressed to enhance cancer detoxifying capability and to neutralize toxic agents during cancer progression [24,25]. The expression of proliferating cell nuclear antigen (PCNA) protein, an essential auxiliary protein for DNA replication processes [26], was then examined as a concomitant event in carcinogenesis. PCNA expression was detected in both the cancerous vicinity—indicated by GST-P positive foci—and the surrounding area. The tested compound can increase cell proliferation in other tissue or surrounding cancerous areas and can act as a mutagen. The apoptotic cells were then detected using the TUNEL assay. Apoptosis is detected and interpreted as the standard endpoint of a chemotherapeutic property of tested compounds, as cancer is eradicated through precisely programmed cell death [27]. However, several reports indicate that apoptotic cells increased during hepatocarcinogenesis (without any chemotherapeutic treatments), which represents a tissue defense against carcinogens [28]. In our case, apoptosis—detected in an early-state of carcinogenesis—refers to the counter mechanism of precancerous cells used to prevent excessive cell proliferation. As such, apoptosis is directly proportional to the progress of carcinogenesis.

Several phytochemical compositions were reportedly found in CP. The potential therapeutic constituents included phenolic compounds, especially xanthone derivatives. Caged-xanthone, pruniflorone U/T, and cochinchinone C were isolated from CP roots [29]. Pruniflorone K/L, cochinchinone A/I, formoxanthone B, braxilixanthone, garcinone B, vieillardiixanthone B, and gerontoxanthone I were also found in CP roots [30,31,32,33], in addition to xanthene and anthraquinone derivatives [18,34]. Dulcisxanthone B from the root displayed anti-inflammation activity in a lipopolysaccharide-activated RAW264.7 cell line by inhibiting nitric oxide production [35], whereas cochinchinone C inhibits cancer phenotypes by downregulating NF-κB in multidrug-resistant A549 lung cancer cell line [36]. The green fruit of CP contains formoxanthone C, pruniflorone M-O, xanthonolignoid, and flavonoid derivative [37,38,39]. Pruniflorone demonstrated potent in vitro nitric oxide inhibitory activity suggesting a potential anti-inflammation property [39], while formoxanthone C induced A549 lung cancer cell death via apoptosis by downregulating histone deacetylase 4 [40]. CP leaves have been reported to be a rich source of phenolic compounds, including flavanone and xanthone. Toxyloxanthone B isolated from the leaves demonstrated an anti-neuroinflammatory activity in the BV-2 microglial cell line and a neuroprotective effect in the SH-SY5Y neuroblastoma cell line [4]. Exudate gum from CP contained terpene (α- and β-pinene) and triterpenoid derivatives [41], which possessed biological activities, including antimicrobial and antioxidant capabilities [42,43]. The stem and bark of CP consisted of several chemical constituents such as epifriedelinol, vismiaquinone B, cochinchinone B, pruniflorone Q/M-R, prunifloroside, and α- and β-mangostin [5,18,19,44]. The methanolic extract of CP wood demonstrated potent free radical scavenging activity [45], and the isolated cochinchinone B triggers several different cancer cells line to die by TNF-α-dependent apoptosis, the antagonizing effect of the NF-κB [44]. Our previous studies [10,15,16] and the other literature confirm that xanthones are major constituents in stem and bark (twig) [15] and that xanthones are responsible for the anti-cancer activities of our extract. Other phytoconstituents in the CP extract could also be contributing to this chemopreventive effect. The whole crude extract could be more potent and safer than its fraction or pure compound [46] due to the synergistic effect of having several constituents in the extract [47]. Similarly, the other phytochemical constituents—such as phenolics and flavonoids—detected in our extract [10] could work synergistically to enhance each other’s efficacy. They may also mask the potent compound’s undesirable effects; supported by the fact that the effective dose (500 mg/kg/BW) displayed no observable side effects.

Multiple signaling pathways are involved in liver tissue’s response to carcinogens which subsequently leads to hepatocarcinogenesis. The major pathways include the signaling of NF-κB (transcriptional factor regulating cytokine production and cell survival) and STAT3 (another transcriptional factor involved in immune/inflammation response and tumorigenesis) [48]. Ethanolic extract of CP [17,49], CP-isolated xanthone and quercetin [8,36] have been reported to attenuate the expression of NF-κB and STAT3 in several cancer cell lines. The cancerous tissues overexpress NF-κB and STAT3 to regulate many downstream genes—promoting cell survival and proliferation—and the crosstalk of these two transcription factors facilitates the establishment of cancer in vivo [48]. In the carcinogen DEN-induced hepatocarcinogenesis model, hepatocyte death induced by DEN releases inflammatory cytokines (e.g., IL-1α) that trigger NF-κB signaling in Kupffer cells. The Kupffer cells then produce a panel of cytokines and growth factors, including IL-6 that further activate STAT3 signaling in hepatocytes, aiming to overexpress critical genes associated with compensatory hepatocyte proliferation survival that subsequently promote liver tumorigenesis [13]. Since the overexpression of NF-κB and STAT3 are also correlated with hepatic inflammation [50], it is possible CP extract be developed for treating inflammation-related diseases (i.e., hepatitis and cirrhosis), as the CP potency was observed in our result accompanied by a reduction in the liver inflammation enzymes ALT/AST.

Excessive and uncontrollable cell proliferation has been recognized as one of the hallmarks of carcinogenesis as several complex mechanisms tightly regulate the process. Cell cycle progression—deploying several cyclins and cyclin-dependent kinases (CDKs)—plays a vital role as the checkpoint for regulating cell phase transition. In hepatic tissue, cell cycle misregulation leads to hepatocarcinogenesis by sustaining excessive cell proliferative signaling [51]. DEN-induced hepatocarcinogenesis reportedly upregulates cyclin D1, a component permitting cell phase progression through the G1 checkpoint [52,53]. CP extract was previously reported to decrease the expression of cyclin D1 and its kinase partner (CDK6) in the hepatoma cell line HepG2 and the breast cancer cell line MCF-7 [20,49,54]. The result agrees with our previous study in which PARP—a downstream effector protein of cyclin D1-CDK6 responsible for DNA repair—was downregulated in HepG2 by CP treatment [15]. A previous study in the cholangiocarcinoma cell line also pointed to CP’s efficacy to decrease the expression of cyclin A and Cdc25; which are responsible for G2 to mitosis (M) phase transition, resulting in G2/M cell cycle arrest [17]. We thus postulated that CP pharmacological activity prevents or retards early-stage hepatocarcinogenesis.

Ours is the first study demonstrating the chemopreventive efficacy of CP against carcinogen-induced hepatocarcinogenesis in rats. CP extract was orally administered and demonstrated a hepatoprotective property against carcinogens with no observable toxicity. The result indicates the effectiveness and safety of CP but also the successful oral bioavailability in vivo. Since most CP extract constituents are water-soluble—evidenced by the extraction process using high hydrophilic solvent [16]—the main constituents after intestinal absorption likely will dissolve primarily in the bloodstream and flood via the hepatic portal vein directly into the liver. The result—it is surmised—will concentrate CP constituents in liver tissue before dilution in the distribution process, facilitating its activity. Furthermore, CP’s hydrophilic constituents are less likely to be affected by the first-pass metabolism, which lowers compound concentrations in the liver, as phase I/II enzymes tend to convert lipophilic to hydrophilic rather than hydrophilic to lipophilic compounds [55]. Further study on bioavailability should be performed to confirm these assumptions.

As for its potential use in humans, the effective dose used in rats (500 mg/kg/week) could be extrapolated to an equivalent human dose of approximately 95 mg/kg/week, using the allometric approach for determining the intraspecies body surface area (K_m__(human, 60kg__)_ = 37 and K_m__(rat, 200g__)_ = 7) [56], which would be 5.7 g/week for a 60 kg human adult. The amount (5.7 g) could be obtained from just 150 g of starting material (yielding 3.78% (*w*/*w*) [16], and CP is widely grown in southeast Asia [57]. There is thus the possibility of developing CP as a health supplement product, considering the following economic factors, including (1) effective amount used; (2) conventional extraction methods with reasonable yields; and (3) accessibility of plant material. The present study presents the in vivo efficacy and safety; however, additional studies are needed to determine CP efficacy and safety and the appropriate dosage and regimen in humans. Further studies should also include pharmacokinetic data of specific compounds (e.g., xanthone and flavonoid) as the representatives of active compounds in CP since the information will be needed for quality control and an internal standard once a product is developed.

## 4. Materials and Methods

### 4.1. Chemicals

Bovine serum albumin, diethylnitrosamine, ethanol, and DMSO were of analytical grade and purchased from Sigma-Aldrich (St. Louis, MA, USA). Rabbit polyclonal GST-placental form (GST-P) antibody was obtained from MBL (Nagoya, Japan). Mouse monoclonal proliferating cell nuclear antigen (PCNA) antibody was obtained from BioLegend (Santiago, CA, USA). EnVision Doublestain system was obtained from Dako (Glostrup, Denmark). Avidin-biotin-horseradish peroxidase complex (ABC) kit was obtained from Vector Laboratories (Burlingame, CA, USA). Apoptosis Detection Kit was obtained from Merck (Cambridge, MA, USA)). Any other chemicals were of analytical grade and were used without any purification.

### 4.2. Materials

CP twig was collected from the northeastern region of Thailand (2013). The voucher specimens (TT-OC-SK-862) were kept at the Medicinal Herbarium, Faculty of Pharmaceutical Sciences, Khon Kaen University, Thailand. The samples were visually authenticated according to taxonomy [58]. A 50% hydroethanolic crude extract was prepared, as previously reported [16]. Briefly, 1 kg of dried twigs was milled and macerated with 6 L of 50% ethanol in water for 7 days. The solvent was then filtered, evaporated (<40 °C), and freeze-dried to get the final crude extract (3.8%yield (*w*/*w*)). The extract was stored at −20 °C in an airtight container and freshly prepared by dissolving in DMSO before use.

### 4.3. Animals and Exposures

Five-week-old male Wistar rats were obtained from the National Laboratory Animal Center, Mahidol University, Nakorn Prathom, Thailand. All rats were housed under controlled conditions (25 ± 1 °C, 50–60% relative humidity, under 12 h light/dark cycle) with pelleted food and water ad libitum. The animal protocol was reviewed and approved by the Animal Ethics Committee of the Faculty of Medicine, Chiang Mai University, Thailand (48/2558) and performed according to the institutional guidelines.

### 4.4. Experimental Design

**Figure 6:**
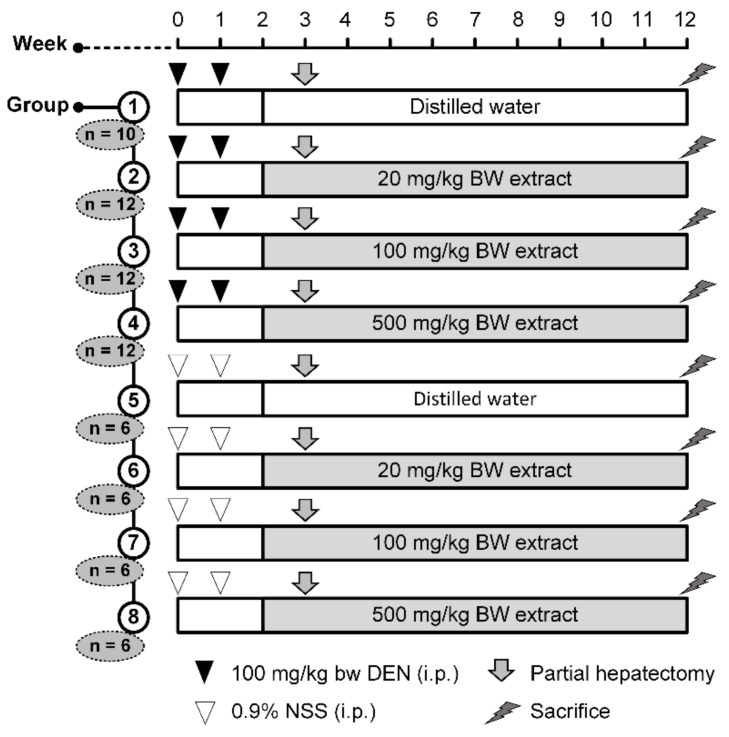
Experimental design to determine the chemopreventive property of CP in DEN-induced rats. BW: body weight; DEN: diethylnitrosamine; NSS: normal saline solution.

Sixty-four male Wistar rats were randomly divided into eight groups and treated as shown in Figure 6. Groups 1 to 4 were intraperitoneally injected with DEN at a concentration of 100 mg/kg BW at the start of the experiment (week 0) and week 1 to induce early-stage hepatocarcinogenesis. Groups 5 to 8 were intraperitoneally injected with normal saline solution (NSS, 4 mg/kg BW) at week 0 and week 1. Group 1, the positive control, was given distilled water orally, while groups 2, 3, and 4 were given 20, 100, and 500 mg/kg BW extract, respectively, from week 2 until the end of the experiment (week 12). Group 5, the negative control, was given distilled water orally, whereas groups 6, 7, and 8 were given 20, 100, and 500 mg/kg BW extract, respectively, from week 2 till week 12. The CP doses were estimated from gallic acid content in CP [16] and daily gallic acid intake in human as described elsewhere [59,60]. All rats were partially-hepatectomized as per Higgins and Anderson [61] to induce hepatocyte proliferation as well as hepatocarcinogenicity. Water/food consumption and body weight were measured twice a week throughout the experiment. At week 12, all rats were sacrificed by exsanguination from the abdominal aorta under isoflurane anesthesia. The livers, spleens, and kidneys were excised and weighed. The liver was cut and fixed in 10% formalin. Three serial sections 4 µm thick were prepared from each liver specimen. One of the serial sections was employed for histological examination after being stained by routine hematoxylin and eosin procedure. The other two sections were used in immunohistochemistry staining as specified below. The remaining liver portions were immediately frozen and kept at −80 °C for further analysis. At the end of the experiment, blood was collected for alanine transaminase (ALT) and aspartate transaminase (AST) activity determination using commercial Olympus kits (Olympus Corp., Tokyo, Japan).

### 4.5. Determination of GST-P Positive Foci 

According to a previous report, immunohistochemical staining for glutathione *S*-transferase placental (GST-P) was performed to determine the preneoplastic lesions in the rat liver tissues [62]. Briefly, the liver tissues were deparaffinized and rehydrated with xylene and ethanol. Hydrogen peroxide (3%) and skim milk (1%) were added to inhibit pseudoperoxidase and to inactivate the nonspecific protein binding, respectively. The samples were then incubated with rabbit polyclonal rat anti-GST-P antibody and antimouse biotinylated antibody. Subsequently, the sample was continually incubated with ABC-PO (rabbit IgG) kit and drenched with diaminobenzidine (DAB). The LAS Interactive measurement program (Leica Microsystems CMS GmbH, Mannheim, Germany) was used to analyze the number and area of GST-P positive foci and any area >0.2 mm^2^ recorded.

### 4.6. Determination of PCNA 

Proliferating cell nuclear antigen (PCNA) was used as a cell proliferation biomarker. PCNA was determined in liver tissues using the EnVision Doublestain system [62]. Liver sections were incubated with citrate buffer (98 °C, 10 min) and H_2_O_2_ then with dual endogenous enzyme block followed by anti-PCNA antibody and polymerase/horseradish peroxidase. The samples were then soaked with DAB. The presence of PCNA protein was confirmed by a brown color in the hepatocyte nucleus. Double stain block was added, followed by rat GSTP polyclonal antibody, rabbit/mouse link, and polymer/alkaline phosphatase (AP) (EnVision System kit). Permanent red was used as the AP substrate. The presence of GST-P was confirmed by a reddish color in the cytoplasm of the hepatocyte. Using a light microscope, we counted the PCNA positive hepatocytes labeled in the GST-positive foci and the surrounding area.

### 4.7. Determination of Apoptotic Cells by TUNEL Assay

Terminal deoxynucleotide transferase-mediated X-dUTP nick-end labeling (TUNEL) assay was used to identify apoptotic cells in liver sections using an ApopTaq peroxidase in situ kit according to the previous report [28]. The samples were deparaffinized, rehydrated, and pretreated with proteinase (20 µg/mL) and H_2_O_2_ (3%), respectively. The samples were incubated in equilibrium buffer for 5 min, and working-strength terminal deoxynucleotidyl transferase (TdT) enzyme was added and incubated for an additional 1 h at 37 °C. The liver sections were treated with an anti-digoxigenin antibody, conjugated with peroxidase, to generate color when exposing to DAB. The numbers of TUNEL positive cells were counted, and the apoptotic index was assessed as apoptotic cells per 1000 hepatocytes.

### 4.8. Statistical Analysis

Values were expressed as group means ± SD. All results were analyzed for normality of distribution (Shapiro–Wilk test) and homogeneity of variance (Levene’s test). The data with a normal distribution (*p* > 0.05, Shapiro–Wilk and Levene’s test)—including measurements of rat body and internal organ weight, as well as ALT/AST expression—were further analyzed using one-way ANOVA followed by the least-significant difference (LSD) post hoc test. The data with a non-normal distribution—which included the measurements of GST-P positive foci, PCNA, and apoptotic cells as indicated by *p* ≤ 0.05, Shapiro–Wilk and Levene’s test—were further determined using the nonparametric Mann–Whitney U test. All statistical analyses were performed using SPSS 19.0 for Window^®^ (SPSS Inc., Chicago, IL, USA). Differences were considered statistically significant at *p* ≤ 0.05 (parametric LSD’s post hoc or nonparametric Mann–Whitney U test).

## 5. Conclusions

In this study, we demonstrated the chemopreventive property of *Cratoxylum formosum* ssp. *pruniflorum* (Kurz) Gogelein in vivo. DEN induced early-stage hepatocarcinogenesis in rats, and subsequent formation of cancerous tissues was detected using immunohistochemical procedures. CP caused no observable toxicity in the regimen used based on the fact that there was no significant difference among treatment groups vis-à-vis body weight, vital organ weight, or consumption of food/water. The hepatic inflammation induced by DEN was also reduced, as indicated by the lower serum AST level after CP treatment. The expressions of GST-P positive foci, PCNA, and apoptotic cells were increased after initial DEN injection, indicating the occurrence of early-stage hepatocarcinogenesis. The oral administration of CP—especially at 500 mg/kg BW—decreased these expressions (viz., GST-P positive foci, PCNA, and apoptotic cells), thereby exhibiting a chemopreventive effect against DEN-induced hepatocarcinogenesis. Our results indicate the efficacy of CP for the prevention of hepatocarcinogenesis. Further safety, toxicity, and clinical in vivo studies will be needed to confirm its potential cancer-preventive character.

## Figures and Tables

**Table 1:** Effect of CP on male Wistar rat body weight (BW) and food/water consumption.

Group	Treatment *		Initial Weight (g)	Final Weight (g)	Food Consumption (g/rat/day)	Water Consumption (g/rat/day)
	Initiator	Test Compound				
1	DEN	DW	206 ± 12	434 ± 52	22 ± 1	32 ± 4
2	DEN	CP 20 mg/kg	209 ± 7	438 ± 41	21 ± 2	27 ± 2
3	DEN	CP 100 mg/kg	205 ± 8	394 ± 43	22 ± 2	28 ± 3
4	DEN	CP 500 mg/kg	205 ± 9	393 ± 24	21 ± 2	26 ± 3
5	NSS	DW	211 ± 7	446 ± 21	23 ± 4	29 ± 9
6	NSS	CP 20 mg/kg	203 ± 8	447 ± 35	23 ± 4	31 ± 3
7	NSS	CP 100 mg/kg	208 ± 8	448 ± 33	21 ± 1	30 ± 3
8	NSS	CP 500 mg/kg	204 ± 10	465 ± 32	25 ± 3	30 ± 7

* DEN (10 mg/kg BW) or NSS (4 mL/kg BW) initially administered at weeks 0 and 1 then either DW (distilled water, 4 mL) or CP (20, 100, and 200 mg/kg BW) fed weekly starting from week 2 until the end of the experiment (week 12).

## Data Availability

The data presented in this study are available from the corresponding authors upon reasonable request.

## Sample Availability

Samples of *Cratoxylum formosum* ssp. *pruniflorum* extract is available from the corresponding author (N.W.).
